# Dopamine and Acetylcholine in the Striatum: Circuit Interactions and Behavioral Control in Substance Use Disorders

**DOI:** 10.3390/brainsci16040397

**Published:** 2026-04-07

**Authors:** Oyku Dinckol, Noah H. Wenger, Aryanna Copling, Bhumiben P. Patel, Munir Gunes Kutlu

**Affiliations:** 1 Center for Substance Abuse Research, Lewis Katz School of Medicine, Temple University, Philadelphia, PA 19140, USA; oyku.dinckol.perni@temple.edu (O.D.); noah.wenger@temple.edu (N.H.W.); bhumiben.patel@temple.edu (B.P.P.); 2 Biomedical Sciences Graduate Program, Lewis Katz School of Medicine, Temple University, Philadelphia, PA 19140, USA; aryanna.copling@temple.edu; 3 Department of Neural Sciences, Lewis Katz School of Medicine, Temple University, Philadelphia, PA 19140, USA

**Keywords:** dopamine, acetylcholine, striatum, nicotine, cocaine, addiction

## Abstract

**Highlights:**

**What are the main findings?**

- Dopamine signaling in the striatum regulates reinforcement learning, motivation, and habit formation.
- Cholinergic interneurons dynamically regulate dopamine release through nicotinic and muscarinic receptors.

**What are the implications of the main findings?**

- Local dopamine–acetylcholine interactions shape striatal circuit plasticity and behavioral flexibility.
- Dysregulation of dopamine–acetylcholine signaling contributes to addiction-related behaviors and SUD vulnerability.

**Abstract:**

Substance use disorder (SUD) is a chronic neuropsychiatric condition characterized by persistent drug seeking and impaired behavioral control. Dopaminergic signaling has long been recognized as a central regulator of reinforcement learning, motivation, and habit formation. Addictive substances profoundly alter dopamine transmission through multiple mechanisms. These drug-induced changes contribute to the initiation, escalation, and persistence of addictive behaviors. In addition to dopamine, the cholinergic system has emerged as an important modulator of striatal circuit function. Acetylcholine and its receptors interact extensively with dopaminergic pathways, shaping striatal signaling dynamics and influencing learning and action selection, with particularly strong relevance for nicotine dependence. In this review, we discuss how striatal dopamine and acetylcholine contribute to learning, habit formation, and addiction-related behaviors, as well as how these systems interact at the circuit level. By integrating these findings, we propose a framework for understanding how dopamine–acetylcholine interactions may influence behavioral regulation relevant to substance use disorders.

## 1. Introduction

The brain’s reward and reinforcement circuits play a central role in guiding adaptive behavior by linking environmental stimuli with motivational value and action selection [1,2,3]. These processes rely heavily on neuromodulatory systems, particularly dopaminergic and cholinergic signaling within the striatum, which regulate learning, motivation, and behavioral flexibility [4,5]. Because these circuits determine how rewards are learned and pursued, they are particularly vulnerable to dysregulation by addictive substances. Substance use remains one of the most pressing public health challenges worldwide, contributing substantially to morbidity, mortality, and socioeconomic burden [6]. Repeated exposure to addictive substances can lead to substance use disorder (SUD), a chronic neuropsychiatric condition characterized by compulsive drug seeking, persistent use despite negative consequences, and a high propensity for relapse [7,8]. Clinically, SUD unfolds through recurring cycles of acute drug exposure, withdrawal, abstinence, and craving, reflecting profound disruptions in the neural systems that govern motivation, learning, and behavioral control [8,9].

Decades of research demonstrate that addictive substances induce long-lasting adaptations in brain circuits responsible for reward processing, decision-making, and memory [9,10]. These circuit-level changes progressively bias behavior toward immediate reinforcement while weakening cognitive control mechanisms, ultimately promoting compulsive drug use [11,12,13,14]. Importantly, distinct neural processes contribute to different stages of the addiction trajectory, with many converging on striatal pathways embedded within the broader dopaminergic system [8,9,15,16]. Dopamine (DA) has long been recognized as a central mediator of reinforcement learning, motivation, and behavioral adaptation [17,18,19]. Through its widespread influence on neural circuits that encode value, prediction, and action selection, dopaminergic signaling plays a critical role in shaping both adaptive and maladaptive behaviors [20,21]. Dysregulation of this system is a defining feature of substance use disorders [14,22,23,24], highlighting DA as a core substrate of addiction-related neuroplasticity.

DA does not operate in isolation. Increasing evidence highlights the importance of interacting neuromodulatory systems that shape dopaminergic signaling and striatal circuit dynamics [25,26,27,28,29]. Among these, acetylcholine (ACh) has emerged as a critical regulator of neural activity linked to attention, learning, motivation, and action selection, all of which are processes vulnerable to disruption in SUD [30,31,32,33,34]. However, the dynamic interplay between dopaminergic and cholinergic signaling remains incompletely understood, particularly in the context of chronic drug exposure, highlighting an important area for continued investigation. Here, by integrating molecular, circuit, and behavioral perspectives, we discuss how dopaminergic and cholinergic systems contribute to processes relevant to SUDs and how their interactions at the circuit level may shape behavioral regulation.

## 2. Dopaminergic Regulation of Cognitive Function and Addiction-Related Behaviors

### 2.1. Dopaminergic System Organization and Receptor Expression

DA embodies the majority of the central nervous system catecholamines by 80% [16,35], and the vast majority of DA neurons are located in the ventral midbrain, namely the ventral tegmental area (VTA) and substantia nigra (SN) [29,36]. The midbrain dopaminergic system connects to three main hubs: the cortex, ventral striatum (nucleus accumbens), and dorsal striatum, forming mesocortical, mesolimbic, and nigrostriatal pathways, and widely distributing dopaminergic receptors across these three areas [16,37,38,39,40,41]. DA receptors (DRs) are members of the seven transmembrane domain G protein-coupled receptor family [42,43,44]. There are five types of DA receptors: D1, D2, D3, D4, and D5, which are known to be involved in different functions [16]. These DR subtypes are classified under “D1-like DRs” and “D2-like DRs” based on their structural, pharmacological, and biochemical properties. Members of these subfamilies have a common homology of transmembrane proteins. The D1-like DR subfamily consists of D1 and D5, whereas the D2-like subfamily consists of D2, D3, and D4. D2-like receptors display approximately 10–100-fold higher DA affinity than D1-like receptors [44,45]. This disparity suggests that receptor recruitment is strongly dependent on extracellular DA concentrations, potentially enabling differential engagement during tonic versus phasic signaling. A primary distinction between these receptor classes lies in their coupling to distinct G-protein subtypes. D1-like receptors stimulate cyclic AMP (cAMP) production through activation of Gαs/olf proteins, whereas D2-like receptors inhibit adenylyl cyclase via Gαi/o signaling [43,46]. DA receptors can form heteromeric complexes and engage non-canonical intracellular pathways, suggesting that downstream effects cannot be explained solely by cAMP modulation [45,47]. Together, this anatomical and molecular organization positions DA as a central regulator of behavioral adaptation. This organization reflects a highly complex system shaped by diverse inputs from multiple brain regions and a heterogeneous receptor architecture that enables flexible modulation of neural activity and behavior.

### 2.2. Dopaminergic Mediation of Cognitive Function

DA transmission within the mesolimbic and mesocortical pathways is involved in several cognitive processes, including attention, learning, memory, motivation, and decision-making [17,18,19]. Furthermore, the dopaminergic system mediates working memory, an important component of cognition, as shown in rodents [48,49], non-human primates [48,50,51,52,53], and humans [54,55,56,57,58,59]. DA is also a central regulator of motivation [60,61], reinforcement learning [62,63,64,65], reward processing [17,66], and contributes to the attribution of incentive salience to environmental stimuli [2]. In addition, dopaminergic signaling plays an important role in regulating goal-directed behavior by influencing action selection and the evaluation of expected outcomes, while also contributing to the gradual emergence of habitual behaviors during repeated reinforcement [4,67,68]. In addition to these cognitive and motivational roles, studies have shown that lesions of dopaminergic pathways reduce movement initiation and execution, and that DA depletion is associated with diseases such as Parkinson’s Disease (PD) [16,17,20]. The current treatment strategy for PD is DA replacement therapy using L-DOPA administration, a DA precursor [69]. Evidence further suggests that both hypo-dopaminergic state following L-DOPA withdrawal and chronic L-DOPA treatment-induced hyper-dopaminergic state are associated with deficits in cognitive flexibility [70,71,72,73,74,75]. Overall, these results suggest an “inverted U” relationship between DA and cognition [76,77,78,79], which implies an optimal DA range for different cognitive processes.

Although DA has traditionally been linked to reward and pleasurable experiences, accumulating evidence indicates that its role is better characterized as signaling prediction error, salience, and learning rather than reward itself [80]. In the field of dopaminergic information encoding, the dominant theory has been the Reward Prediction Error (RPE) Hypothesis. The RPE hypothesis posits that midbrain DA neurons respond positively to surprising (unexpected) rewards (e.g., by increasing firing frequency) [3,15,17,80]. This DA response moves back to the time of cues that predict the reward. However, recent studies indicate that DA release in NAc core terminals may diverge from this pattern and is influenced by the novelty and saliency of external stimuli and non-reward outcomes [81,82,83], as well as by signaling the value of work/reward [84,85], suggesting that DA may function outside the boundaries of reward prediction.

Furthermore, recent computational frameworks have refined this view. The distributional reinforcement learning framework proposes that dopaminergic populations encode a distribution over predicted returns rather than only the expected value [86,87]. In contrast, the retrospective causal learning framework (also known as ANCCR) suggests that DA signals retrospective causal credit assignment, evaluating which preceding events are responsible for observed outcomes. Thus, ANCCR proposes to capture DA ramps shaped by the environmental timescale, where DA activity gradually increases as an animal approaches an expected outcome, reflecting learned temporal structure and uncertainty in reward prediction [88,89,90]. Collectively, these findings support the view that DA serves as a dynamic neuromodulatory signal that integrates cognitive, motivational, and behavioral information to guide adaptive decision-making.

### 2.3. Dopaminergic Mediation of SUD-Phenotypes

Since DA critically supports adaptive cognition and goal-directed behavior, disruptions in dopaminergic signaling can profoundly alter behavioral control. Such disruptions are central to the emergence of substance use disorder (SUD)-associated phenotypes, including impairments in social behavior, executive function, and risk evaluation, reflecting maladaptive engagement of neural mechanisms that normally guide learning and action selection [91,92,93,94,95]. As mentioned above, the DA system significantly influences both goal-directed and habitual actions that emerge from reward learning by conveying expectancy, value, and action invigoration. The emergence of SUD-associated phenotypes involves maladaptive changes in these behavioral classifications [12,13].

Two prominent views in the addiction literature propose that substance-induced changes in DA neurotransmission contribute to addictive phenotypes through either reduced (hypoactive) or enhanced (hyperactive) DA signaling within mesolimbic and striatal circuits [96,97]. One view suggests that repeated drug use reduces sensitivity to natural rewards by elevating reward thresholds through a drug-induced hypodopaminergic state. This reduced reward sensitivity may shift behavior toward habitual drug seeking and diminish inhibitory control, promoting continued drug use despite negative consequences [14,96,97,98]. The second view proposes that repeated drug use sensitizes mesolimbic DA signaling, increasing the incentive salience attributed to drug-associated cues and making drug-related stimuli more attention-grabbing and motivationally significant. As a result, drug-associated cues evoke stronger motivational responses, leading to elevated craving and drug-seeking behavior. In this framework, sensitized DA signaling enhances the motivational “wanting” of drug-associated cues without necessarily increasing the hedonic “liking” of the drug itself [2,96,99,100].

Supporting these hypotheses, mesocorticolimbic and nigrostriatal DA transients have been associated with drug-seeking (craving), changes in reward value (e.g., loss of euphoria/anhedonia), and habit formation, respectively; however, these pathways are involved in multiple behavioral processes and are not limited to these functions [16]. As widely shown in the literature, striatal dopaminergic signaling plays an important role in the emergence of drug-seeking (i.e., cravings). For example, cocaine self-administration in rats increases DA efflux in the dorsolateral striatum (DLS) [101] and nucleus accumbens (NAc) [102] during cocaine-seeking and cocaine-associated cues. Likewise, disruptions in dopaminergic signaling via lesions in NAc core [103] and DR antagonist infusion into DLS [100,104] disrupt cocaine-seeking in rats. Furthermore, DA blockade via infusion of selective D1-like and D2-like DR antagonists into NAc core abated incubated cocaine- [105] and methamphetamine- [106] seeking in rats, a time-dependent increase in drug seeking following prolonged withdrawal, indicating the importance of DA in individuals’ vulnerability to relapse. Finally, drug-driven increases in striatal DA are also associated with self-reported reward experiences (i.e., euphoria) [107,108,109].

Beyond its role in reward and craving, striatal DA signaling also contributes to the formation of habitual behaviors [110], a process that has been proposed as one of the mechanisms contributing to the development and persistence of substance use disorders [111,112,113]. From the beginning of drug use to established drug use, striatal phasic DA signaling emerges in the ventromedial striatum (VMS, including the NAc) and then sequentially increases in DLS while it declines in the VMS as drug seeking progressively escalates [114,115]. Temporary inactivation of the DLS decreased habitual drug-seeking in rats [116], suggesting that disruption of the habit system can shift behavior away from habitual responding and back toward goal-directed control. In line with this, neuroimaging studies conducted in monkeys [117] and humans [118] show progressive DLS engagement after prolonged drug use.

Thus, dopaminergic circuitry is heavily involved not only in adaptive learning processes but also in behavioral changes associated with substance use disorders. Accordingly, dopaminergic signaling has been implicated in mechanisms such as habit formation and sensitization to drug-associated cues. Collectively, these findings suggest that striatal DA inputs contribute to the shift from goal-directed to habitual drug-seeking behavior, which may become less sensitive to outcome devaluation or negative consequences, highlighting their important role in the neurobiology of addiction. However, DA alone cannot fully account for the complex circuit adaptations that underlie addiction, pointing to the involvement of additional neuromodulatory mechanisms, including ACh.

## 3. Dopamine–Acetylcholine Circuit Interactions in the Striatum and Their Relevance to Addiction

As discussed above, DA has long been central to understanding reinforcement-related learning and drug-seeking behavior, including shifts toward habitual responding and incubation of drug seeking in SUD. However, growing evidence indicates that DA does not act in isolation. Instead, its functional impact is shaped by interactions with other neuromodulatory systems, particularly ACh. This dopaminergic–cholinergic interplay has emerged as a critical area of investigation within brain regions that govern reward processing, learning, salience detection, and habit formation, including the striatum, and may provide important insight into the circuit mechanisms underlying SUD-related phenotypes.

### 3.1. Cholinergic Signaling: Organizing Principles Within the Striatum

While dopaminergic signaling plays a central role in addiction-related behaviors, it operates within a broader neuromodulatory framework in which ACh serves as a critical regulator of striatal circuit function [30,119,120,121,122,123,124]. Within the striatum, ACh is released predominantly by cholinergic interneurons (CINs), a small yet highly influential neuronal population that exerts widespread control over local network activity [119,122,123]. Through their extensive axonal arborizations and tonic firing patterns, CINs are uniquely positioned to shape information processing, modulate DA release, and influence behavioral output [125,126,127]. Although cholinergic projection neurons originating from regions such as the pedunculopontine and laterodorsal tegmental nuclei may provide additional input [128,129], but converging evidence indicates that local CINs represent the principal source of ACh within the striatum [130]. This anatomical arrangement enables rapid, spatially precise modulation of striatal microcircuits that govern action selection, reinforcement learning, and habit formation, processes that are highly vulnerable to disruption in SUDs.

Cholinergic transmission in the striatum is mediated through two receptor families: muscarinic ACh receptors (mAChRs) and nicotinic ACh receptors (nAChRs) [131]. mAChRs are G-protein-coupled receptors capable of exerting either excitatory or inhibitory effects and account for the majority of cholinergic receptors [132,133]. In contrast, nAChRs are ligand-gated ion channels assembled as homo- or heteromeric complexes of α and β subunits, allowing ACh to rapidly influence neuronal excitability and neurotransmitter release [134]. Together, these receptor classes enable cholinergic signaling to regulate striatal activity across both slow modulatory and fast synaptic timescales. Five muscarinic receptor subtypes (M1–M5) are broadly organized into two functional groups based on G-protein coupling: M1-like receptors (M1, M3, M5), which signal through Gq pathways, and M2-like receptors (M2, M4), which couple to Gi/o proteins [132]. Nicotinic receptors similarly exhibit substantial diversity, consisting of heteromeric assemblies formed by combinations of α (α2–α6, α10) and β (β2–β4) subunits, as well as homomeric receptors typically composed of α7 or α9 subunits [134]. This receptor-level specialization, combined with CINs’ central role in orchestrating local striatal circuit activity, makes cholinergic signaling a critical regulator of striatal DA dynamics.

### 3.2. CIN–Dopamine Interactions

**Figure 1:**
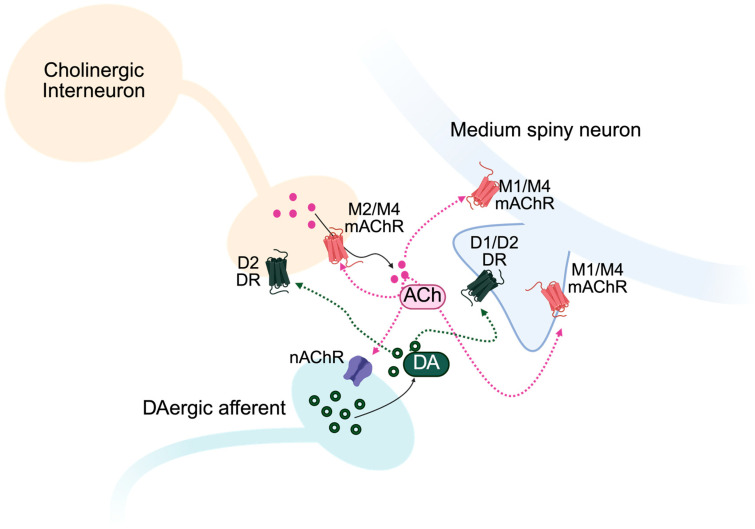
Muscarinic and Nicotinic Control of Dopamine–Acetylcholine Interactions in the Striatum. The interaction between local striatal cholinergic neurons and dopaminergic afferents mediates the activity of medium spiny neurons in the striatum. DA, dopamine; ACh, acetylcholine; mAChR, muscarinic acetylcholine receptor; nAChR, nicotinic acetylcholine receptor; D2, D2 dopamine receptor.

A defining feature of striatal microcircuitry is that DA signaling is shaped not only by midbrain neuronal firing but also by powerful local circuit mechanisms (Figure 1) [135,136]. Among these, CINs, the primary source of ACh within the striatum, serve as key regulators of dopaminergic activity through their dense axonal arborizations [125,137]. CINs are uniquely capable of coordinating network-level activity and dynamically influencing the spatial and temporal structure of DA signaling. This local regulation enables rapid modulation of dopaminergic tone that can be partially decoupled from midbrain activity.

Critically, the dense local connectivity of CINs and their presynaptic control of dopaminergic terminals enable a temporal coordination between cholinergic interneuron activity and DA signaling [28,135,138,139]. This ACh-DA interaction between CINs and DA terminals may vary across striatal subregions, thereby impacting behavioral regulation. Indeed, a recent study demonstrated that the temporal relationship between CIN pauses and dopamine signals differs across dorsal-lateral, dorsal-medial, and ventral striatal circuits, revealing region-specific coupling between cholinergic activity and dopaminergic reward signals [140]. Other striatal circuitries and interactions also mediate these responses and physiological outcomes [26,136,137,141]. Reciprocal interactions between CINs and DA fibers (Figure 1) have been demonstrated across multiple experimental approaches [126,127,142,143]. Intra- and extracellular recordings show that CIN pauses, coinciding with DA transients and medium spiny neuron depolarization, create permissive conditions for DA-dependent synaptic plasticity in the striatum [142]. Importantly, dopaminergic and cholinergic transients are not obligatorily coupled; their temporal relationship can vary depending on behavioral context and circuit state [125,138,144]. Voltammetry studies, combined with in vivo and in vitro optogenetic stimulation, demonstrate that ACh release from striatal cholinergic interneurons can evoke localized DA release in both the dorsal and ventral striatum [126,127].

Together, these findings suggest that DA and ACh signals interact through flexible, context-dependent mechanisms that allow coordinated modulation of striatal activity and behavior [29,126,138,145]. One influential framework describing this complex interaction is the “activator–inhibitor” model, in which cholinergic signaling promotes DA release, while DA feeds back to constrain cholinergic activity [29,146,147,148,149,150,151]. However, growing evidence indicates that this relationship is not strictly reciprocal. CIN responses do not always depend directly on dopaminergic input, as cholinergic and dopaminergic afferents can exhibit distinct sensitivities to reward-related events [125,144]. Accordingly, pharmacological and genetic disruption of dopaminergic signaling in dorsomedial striatum (DMS) reduces, but does not eliminate characteristic CIN pause responses, pointing to the involvement of additional circuit mechanisms [152]. Indeed, excitatory inputs from thalamic and cortical regions have been implicated in regulating cognitive flexibility and action selection by shaping CIN activity [1,153], supporting a modulatory role of DA [28,135,137,138,139,141].

In addition, CIN pause–rebound dynamics provide further insight into how cholinergic signaling influences striatal processing. Following the termination of the CIN pause, a rebound phase marked by increased firing typically ensues [136,154]. This rebound arises from intrinsic membrane properties but is strongly influenced by context-dependent excitatory drive, indicating that CIN output reflects the integration of intrinsic conductance and distributed network inputs [137,144,155,156,157,158]. DA further refines this process by regulating the magnitude and timing of rebound activity, thereby tuning CIN excitability and its downstream influence on striatal processing [26,159,160].

Together, CINs emerge as central coordinators of striatal circuit dynamics. Rather than operating through a simple linear pathway, DA–ACh interactions arise from multilayered network processes in which intrinsic CIN properties and distributed inputs shape neuronal responsiveness and regulate striatal circuit activity.

### 3.3. Receptor Mechanisms Governing Cholinergic Control of Dopamine Release

While circuit-level studies establish that CINs exert powerful control over striatal DA signaling, the mechanisms through which this regulation is implemented arise from specialized receptor systems distributed across cholinergic and dopaminergic elements. Dissecting these receptor-level processes provides critical insight into the molecular logic through which ACh gates DA output and regulates striatal information processing. Broadly, nAChRs provide rapid, temporally precise control over DA release, whereas mAChRs exert slower modulatory influences that shape cholinergic tone and presynaptic excitability.

At the molecular level, cholinergic regulation of DA signaling is mediated by nAChRs located on dopaminergic axons [25,161]. Activation of these receptors directly depolarizes DA terminals, increasing calcium entry and the probability of vesicular release, thereby enabling rapid, spike-independent modulation of DA output [162]. Pharmacological blockade of nAChRs using antagonists such as mecamylamine or DHβE disrupts striatal DA release, highlighting the critical role of axonal nAChRs activated by endogenous Ach [30,32,158,159,163,164]. Because DHβE selectively targets β2-containing nAChRs (e.g., α4β2, α6β2), these findings suggest that nigrostriatal DA axons predominantly express β2-containing nAChRs [30,126,163]. Consistent with this, antagonism of α6-containing nAChRs reduces accumbal DA release, further implicating β2-dependent nicotinic mechanisms in DA gating [165]. Together, these findings indicate that axonal nAChRs provide a powerful mechanism through which striatal cholinergic activity can sculpt the magnitude, timing, and spatial specificity of DA signals during behaviorally relevant states.

Muscarinic receptors modulate striatal DA transmission in an activity- and mechanism-dependent manner: M2/M4 receptors inhibit striatal ACh release and, by reducing nicotinic receptor activation, decrease the nAChR-dependent component of DA release. Administration of non-selective mAChR agonists, such as oxotremorine, produces DA depression via M2/M4 activation in both dorsal and ventral striatum [164,166]. Knockout studies indicate that M4 muscarinic receptors are required for regulating striatal dopamine signaling, whereas M2 receptors appear to play a more restricted role, being necessary primarily in the dorsal striatum for shaping dopamine transients [164]. In line with these findings, non-selective mAChR antagonism with scopolamine produces robust increases in striatal DA release [27,127,166]. M1 and M4 mAChRs are also expressed on MSNs, where they regulate neuronal excitability and corticostriatal synaptic transmission, contributing to cholinergic modulation of striatal output [167,168,169]. Notably, these receptors show cell-type-specific expression patterns across striatal projection neurons. M1 receptors are more prominently expressed on D2 MSNs, whereas M4 receptors are enriched on D1 MSNs, suggesting that muscarinic signaling can differentially modulate the direct and indirect pathways of the basal ganglia [167,168].

Together, these findings demonstrate that cholinergic control of striatal DA release is implemented through a finely organized receptor expression that enables both rapid and sustained modulation of dopaminergic signaling. ACh regulates DA transients through multiple receptor mechanisms: nAChRs located on DA terminals provide rapid, temporally precise gating of DA release, whereas mAChRs exert complementary modulation by shaping cholinergic tone and presynaptic excitability. This complementary receptor organization (fast nicotinic receptor control of DA release and slower muscarinic modulation of cholinergic tone and presynaptic excitability) allows ACh to tune DA output across multiple timescales, supporting flexible regulation of striatal information processing. Such receptor-level mechanisms provide a critical molecular foundation for understanding how cholinergic–dopaminergic interactions influence learning, plasticity, and vulnerability to maladaptive behavioral states.

### 3.4. Behavioral and Addiction Relevance

While receptor- and circuit-level studies provide critical insight into how ACh shapes dopaminergic signaling, the functional significance of this interaction is most clearly revealed during behavior. Neuromodulatory coordination between ACh and DA plays a central role in learning, action selection, and reinforcement processes that guide adaptive behavior. Consistent with this framework, coincident dopaminergic and cholinergic responses have been observed during learning [125,170], yet ACh transients can also occur independently of DA depending on task contingencies. For example, during reward omission, ACh signaling in the putamen and NAc, as well as in the ventrolateral portion of the dorsal striatum, persists despite reduced dopaminergic activity or DA receptor loss, whereas during reward or reward-predicting cue presentation, ACh responses are often DA-dependent [125,138,152]. Together, these findings support the idea that ACh-DA coupling varies across behavioral contexts and task structures.

This striatal ACh-DA coupled interaction plays a significant role in SUD as it modulates behavioral processes that are dysregulated with substance abuse, including reinforcement learning, cue-driven motivation, and value determination of rewards, behavior flexibility, habit formation, and habitual or compulsive drug seeking, as well as drug reinforcement and withdrawal-related adaptations [32,171]. Early studies demonstrated that addictive drugs shift the balance between these neuromodulators: apomorphine and amphetamine increase ACh content in striatal tissues via DA receptor activation [172,173], while morphine withdrawal is associated with reduced DA release and increased ACh output [174]. Ethanol-induced accumbal DA release is attenuated by blockade of mAChRs or nAChRs and by CIN depletion, indicating that intact cholinergic signaling is required for ethanol’s dopaminergic effects [27]. Similarly, deletion of D2 receptors from CINs attenuates the reinforcing properties of cocaine in knockout models [175]. Collectively, these observations support the notion that cholinergic signaling operates in concert with dopaminergic transmission to dynamically regulate striatal circuit function, and that disruption of this interaction may contribute to maladaptive learning, habit formation, and the emergence of addiction-related behaviors.

## 4. Conclusions

The evidence reviewed here indicates that coordinated interactions between dopaminergic and cholinergic signaling in the striatum do not simply accompany each other but may influence striatal circuit dynamics by modulating local transient timing and magnitude. Taking the behavioral impact of striatal ACh-DA neuromodulatory interaction into account, addiction cannot be fully understood through the lens of either system in isolation. Rather, SUD-related phenotypes may arise from disruptions in the coordination among these neuromodulatory networks, where ACh is positioned to dynamically influence DA release, synaptic plasticity, and circuit-level information processing.

Across molecular, cellular, circuit, and behavioral levels of analysis, a convergent picture is emerging that cholinergic interneurons function as important modulators of dopaminergic signaling, shaping striatal circuit dynamics. However, this interaction may not operate uniformly across the striatum and may vary across striatal subregions. In ventral domains of the striatum, particularly the nucleus accumbens, ACh-DA interactions have been associated with reinforcement-related processes. In contrast, in dorsal striatal domains, these interactions have been linked to context-dependent modulation of behavioral responses.

The bidirectional nature of ACh-DA signaling introduces multiple points of vulnerability, but also potential opportunities for therapeutic intervention in SUD. Pharmacological and genetic studies suggest that targeting receptor systems governing this interaction, including β2-containing nicotinic receptors and muscarinic receptor subtypes, may offer greater specificity than approaches focused solely on DA transmission. Such strategies may help restore neuromodulatory balance rather than broadly suppress reward circuitry, a limitation that has historically constrained addiction treatments.

In sum, the current literature suggests that locally gated, subregion- and context-dependent dopamine–acetylcholine interactions shape striatal signaling and may influence behavioral processes relevant to substance use disorders, including reinforcement-related learning, cue-driven motivation, and drug-seeking behavior.

## Data Availability

Data sharing is not applicable.

## Abbreviations

The following abbreviations are used in this manuscript:

SUD	Substance Use Disorder
ACh	Acetylcholine
DA	Dopamine
VTA	Ventral Tegmental Area
SN	Substantia Nigra
DR	Dopamine Receptor
cAMP	Cyclic Amp
PD	Parkinson’s Disease
RPE	Reward Prediction Error
NAc	Nucleus Accumbens
DLS	Dorsolateral Striatum
VMS	Ventromedial Striatum
CIN	Cholinergic Interneuron
mAChR	Muscarinic Acetylcholine Receptor
nAChR	Nicotinic Acetylcholine Receptor
